# What Makes Chinese Adult Children Behave Differently during Parents’ End of Life: A Discriminant Analysis of Macao Chinese

**DOI:** 10.3390/ijerph182010737

**Published:** 2021-10-13

**Authors:** Wai I. Ng, Sok Leng Che, Xiang Li, Ming Xia Zhu

**Affiliations:** 1 Education Department, Kiang Wu Nursing College of Macau, Macao SAR 999078, China; shirleyli@kwnc.edu.mo (X.L.); zmx@kwnc.edu.mo (M.X.Z.); 2 Nursing and Health Education Research Centre, Kiang Wu Nursing College of Macau, Macao SAR 999078, China; shirley@kwnc.edu.mo

**Keywords:** filial piety, end-of-life, general public, Macao, Chinese, discriminant analysis

## Abstract

The daily practice of filial piety (FP) is well prescribed under the traditional filial norms in the Chinese community. However, exploration of FP practices at the end of parents’ lives is limited. The current study explored the FP representation and good death preferences of Macao Chinese. A cross-sectional web-based survey was conducted and discriminant analysis was used to identify possible predictors of FP representation in the context of parents’ end of life. Results showed that Macao Chinese were inclined to perform most of the filial duties in the last journey of their parents. Among 705 participants, 150 (21.3%) tended to practice authoritarian FP, and 555 (78.7%) tended to practice reciprocal FP. Age, education, religion, and good death preferences were identified as predictors of different FP representation groups. The findings could help clinicians to obtain a preliminary perception of FP representation of Chinese patients and to determine the appropriate approach for end-of-life care from a family perspective.

## 1. Introduction

Filial piety (*Xiao*; FP), the central virtue of Confucianism, is the dominant social and family value and norm applied in most, if not all, societies sharing the Chinese culture, which regulates and sets the socially acceptable standard of behaviour for children toward their parents. Over the past several decades, the concept of FP has undergone historical development. Yeh [1] divided the concept by introducing the dual filial piety model (DFPM) which comprises two aspects of FP, namely the authoritarian FP and reciprocal FP. Authoritarian FP emphasizes hierarchy and submission to authority, which entails obedience, loyalty, and reverence from children to parents at all costs based on seniority in the family. For example, the communication between parents and children is likely through one-way conversation rather than an interactive manner, and children usually fulfill the filial duties with a lack of understanding and mutual decision-making under the shadow of hierarchical authority. In contrast, reciprocal FP accentuates interpersonal relationships and the obligation of children to care emotionally and spiritually in order to repay their parents’ efforts in foster care [2,3].

The practice of FP may extensively influence the quality of end-of-life care received by the parents in Chinese society because children usually speak for their parents and regard it as their filial responsibility. Under such circumstances, instead of upholding respect for autonomy that is globally recognized in biomedical ethics [4], many Chinese adult children choose to request the physician to conceal life-threatening conditions from their parents to protect them from “harm” by the bad news [5,6,7,8]. Accordingly, some parents are not able to participate in end-of-life planning and decision-making, or even pass away without being informed about their diagnosis [8,9], which leads to two main ethical concerns: (1) The final wish of parents may not be properly addressed and (2) Clinicians, especially with a non-Chinese ethnicity, may often be forced to encounter a moral conflict between patient autonomy and familial involvement in life and death decisions [6,8,10,11]. These situations have to some extent disregarded the ethical principle of respecting the autonomy and beneficence of the parents, and these situations are unable to be ethically justified in the Western perspectives. Moreover, these ethically challenged situations may seriously hinder the implementation of quality end-of-life care and break the parents’ hope of achieving a good death. A good death is the greatest blessing eventually received by individuals. From the Chinese perspective, a good death is commonly perceived as “a result of natural causes such as aging with a content life and no outstanding life regrets” [12]. However, considering the Chinese FP emphasizes etiquette before and after death [13], the attention paid to dealing with the dying process per se is surprisingly scarce. Moreover, the topic of death is regarded as taboo or a curse between parents and children [14,15]. Findings from different studies suggest that Chinese patients, their families, and clinicians usually struggle with a dilemma over the decision to disclose adverse prognosis, obtaining advanced directives, and begin palliative care, as all these common end-of-life practices are often considered as “disrespectful” or not complying to FP among Chinese families [7,8,10,11]. China, as the most populous country in the world for the past decade, has accounted for close to one fifth of the global total population according to the World Population Prospects 2019 [16]. In addition, it is estimated that there are around 50 million Chinese living in all over the world [17]. For example, there were 5.4 million Chinese Americans in the United States, making up the largest Asian origin group in the country [18]. Therefore, understanding the core value of FP and its influences on end-of-life decision-making among Chinese families are crucial for clinicians, in order to provide culturally appropriate care for such a large population in the world.

To date, several studies have evaluated the impact of general filial attitudes, filial responsibilities, and filial expectations of children and parents with a Chinese background [13,19,20,21,22,23,24,25]. However, these studies did not explore the association between filial representation or behaviours and good death preferences, nor examined the factors influencing end-of-life filial representations. In addition, the way that the DFPM attributes to the end-of-life filial piety representations of the people is still elusive.

There have been prominent and rapid changes in the traditional notion of FP due to exposure to Western culture, religion, and value systems and the evolution of Chinese culture itself [13,25,26]. Macao, as a special administrative region of China, is comprised of mainly Chinese population and is also facing these cultural changes. To what extent the Macao Chinese still adheres to the values of traditional FP, and to what extent DFPM can explain FP representations in the aspect of end-of-life situations amongst Chinese adult children remains uncertain. The aims of this study were to explore the filial representation and good death preferences of Macao Chinese and to identify predictors that allow differentiation of the dual aspects of FP in the context of parents’ end of life. It is anticipated that insights would be provided for understanding the FP representations and FP attributes toward an end-of-life parent among Macao Chinese and subsequently provide valuable evidence for clinicians of all settings to deliver culturally sensitive treatment and care.

## 2. Materials and Methods

### 2.1. Study Design and Participants

This study was a cross-sectional web-based online survey conducted in Macao from December 2020 to January 2021. Participants who were (1) Macao Chinese residents aged from 18 to 74 years and (2) able to give informed consent and understand the questionnaire content were included in this study. Convenient and snowball sampling methods were adopted, invitation for the study was posted on various social media and communication tool, including Facebook, WeChat, and WhatsApp. Participants were encouraged to forward the study invitation on their personal social media platforms to attract wider potential participants. Ethical approval was obtained from the Research Management and Development Department of a Higher Education Institute in Macao (approval number: 2020JAN03).

### 2.2. Instrument and Data Collection

A structured questionnaire was developed and it included the following four sections:

(1) Filial Piety Representations at Parents’ End-of-Life Scale (FPR-EoL): This scale was specially developed and validated for this study. Based on the DFPM, it was constructed with reference to the measurements of FP behaviours [1,3,27,28] and end-of-life-related measures [20,21,29,30] for the Chinese population. It comprises 19 items, and each item indicates an FP representation that adult children would perform during the end of life of their parents. The FPR-EoL scale is a five-point Likert scale, ranging from 5 (“definitely will do”) to 1 (“definitely will not do”). The total score ranges from 19 to 95, with a higher score indicating a higher tendency to perform FP representations. The reliability of this scale was sufficient with Cronbach’s alpha at 0.73. Items of FPR-EoL were assigned to either authoritarian FP or reciprocal FP groups according to DFPM. The mean score of each FP group was calculated, and those with higher scores on specific groups were assigned as people with that type of representation.

(2) Good Death Inventory: Good death inventory (GDI) was developed by Miyashita et al. (2008) and was originally designed for evaluating a good death from the perspective of bereaved family members [31]. It is a self-administered scale translated into Korean, simplified Chinese, and traditional Chinese, and widely used in studies conducted in Asian regions [29,32,33]. GDI consists of 18 domains comprised of 54 good death attributes, and it is measured on a seven-point Likert scale (from 1 = “absolutely disagree,” to 7 = “absolutely agree”). The total score ranges from 54 to 378. The traditional Chinese version of the GDI was selected and approved to be used to investigate the good death preference of Macao general public. The traditional Chinese version demonstrated very good reliability (Cronbach’s alpha = 0.96) [29]. Cronbach’s alpha of the GDI in this study was 0.91.

(3) Socio-demographic information includes gender, age, education level, religion, marital status, employment status, whether the participant had children and siblings, living with parents, whether parents were alive, and closeness of participants with parents.

(4) Health status: including (i) self-rated health measured on a five-point Likert scale from “very bad” to “very good” and (ii) “Do you have any disease that requires regular follow-up?” Participants were to answer “yes” or “no.”

A plain language invitation was provided along with the survey link to explain the aims of the study, the right to participate, and participant anonymity. Having obtained approval from two day centers for older adults, face-to-face interviews were performed by trained investigators with individuals aged 60 or above who may find it difficult to attend an online survey.

### 2.3. General Statistics and Discriminant Analysis

The Statistical Package for the Social Sciences Version 22 (SPSS, v22) was used for data analysis. Descriptive analyses were performed on socio-demographic data, perception of a good death, and the FPR-EoL scale. The Chi-square test and independent sample t-test were performed to examine FPR-EoL differences between socio-demographic variables and GDI. Fisher discriminant modeling was used to perform discriminant analysis.

Fisher discriminant analysis (FDA) was applied to predict a categorical dependent variable (reciprocal and authoritarian FP) using predictor variables (socio-demographic variables and GDI). The FDA determines linear combinations of predictor variables that provide the best discrimination between the groups of dependent variables [34,35]. The discriminant functions are shown in the following equation:

(1) $$d_{ik}=b_{0k}+b_{1k}x_{i1}+\ldots +b_{jk}x_{ij},$$

where *d_ik_* is the value of the *k*th discriminant function for the *i*th case, *p* is the number of predictors, *b_jk_* is the value of the *j*th coefficient of the *k*th function, and *x_ij_* is the value of the *i*th case of the *j*th predictor. The functions were generated from the allocated group membership according to the assigned FP representation groups. A stepwise forward analysis method was used to identify the possible variables for optimal discrimination. Because the distribution of FPR-EoL is different, the prior classification probability was set to compute from the group sizes. The threshold for statistical significance was set at *p* < 0.05.

## 3. Results

### 3.1. Characteristics of the Participants

A total of 1222 individuals representing the Macao general public participated in the survey, out of which, 705 questionnaires (57.69%) were completed and validated, incomplete questionnaires were excluded. Among all participants, over 80% were female, the mean age was 42.04 years (range 18 to 74, *SD* = 15.33), nearly 75% received higher education, and over half were not religious. Over 60% of the participants were not living with parents. Most of the participants had not experienced their parents’ deaths. Among those who had experienced their parent’s death, only a quarter and one-third of the participants were satisfied with the dying process of their father and mother, respectively (Table 1).

Out of the 705 valid responses, 150 were classified as having an authoritarian FP representation when assuming their parents were at the end of their lives, and 555 were classified as having reciprocal FP representation. Participants had higher mean scores on the reciprocal subscale than on the authoritarian subscale, indicating that participants in this study perform more reciprocal representations when their parents were at the end-of-life (Table 1). Under the same circumstances, participants who were aged between 55 and 74, had an education level of high school or below, had marital experience, had Buddhist beliefs, were not employed, had children, and those whose parents were not alive had a higher proportion of having authoritarian representation (Table 2).

### 3.2. Perception of a Good Death

The GDI results showed that the average total score was 305.48 (*SD* = 26.17). The top three domains attributed to a good death in our sample were “Being respected as an individual” (*M* = 19.14, *SD* = 2.04), “Good relationship with family” (*M* = 19.07, *SD* = 2.16), and “Preparation for death” (*M* = 19.04, *SD* = 1.96). (Table 3). A higher score in GDI indicates a better perception of death.

### 3.3. Discriminant Analysis

A classification system was developed using the socio-demographic data and GDI scores of 686 participants as the discriminant variables. The FDA produced a model that met the assumptions for this analysis method. Box’s M test was not statistically significant (Box’s M = 11.53, *p* = 0.33), implying that the variables used in the model were normally distributed [31]. The canonical correlation for this analysis was *r* = 0.29, with an eigenvalue of 0.09, indicating that the discriminating variables explained less variance in the dependent variable. The effect size of our discriminant analysis was r^2^ = 0.09, which had a small effect. Wilks’ Lambda of discriminant function was 0.92, *χ*^2^(4, *n* = 686) = 60.36, *p* < 0.001, which indicates a significant difference between the FP groups. The structure matrix was examined to define the discriminant functions. Age, education, religion, and total GDI score were recognized as predictor variables for the two FP representation groups. The standard forms of the discriminant function equations are as follows:

L1= −71.605 − 0.005 × age + 1.448 × education − 2.642 × religion + 0.463 × GDI,(2)

L2= −72.637 − 0.471 × age + 2.240 × education − 3.148 × religion + 0.471 × GDI(3)

Overall, 56.35% of the participants were accurately classified. The classification function accuracy was 14.7% and 98.0% for the authoritarian and reciprocal FP groups, respectively.

## 4. Discussion

This study revealed that more Macao Chinese were likely to perform reciprocal FP compared to authoritarian FP during the last journey of their parents. Such allegiance to filial duties reflects a bottom-line obligation of children for their parents [36]. Higher filial responsibility was found to be associated with worse self-rated health [37]. This could be attributed to the implementation of more filial duties that may be burdensome for the children and lead to burnout [11,38]. Empirical studies have revealed that Chinese people generally consider the provision of direct bedside care, being responsible for medical expenses, decision-making of treatment for critically ill parents, and forgoing their own wishes to satisfy parents’ FP representations toward end-of-life parents [6,39,40]. Most of these perceived FP representations possess the characteristics of authoritarian FP in terms of the FPR-EoL; conversely, more Macao Chinese were likely to practice reciprocal FP, as demonstrated by our findings. Yeh and Bedford [3] and Sun et al. [22] regarded this as the outcome of the global interaction of cultural exchange, resulting in a shift from conservative authoritarian FP to an interactive reciprocal FP. The dual properties of the FP representations were further recognized in Chinese adult children in the context of the parents’ end of life.

Moreover, age, education, religion, and GDI were significant predictors for conceptualizing authoritarian FP and reciprocal FP. Authoritarian FP emphasizes hierarchy and respecting parents obediently, however, adult children of various age groups may perceive this obligation differently as they belong to different generations. Studies have demonstrated that although older Chinese hold a strong belief in FP, they clearly expressed fewer expectations of filial duties from their adult children because the rapid social changes may lead to a lack of understanding of Confucian FPs in the younger generations [13,26,41]. Therefore, although they were committed to their older parents, they did not expect the same from their children [13]. Hence, compared with younger Chinese, the older Chinese were more likely to practice authoritarian FP.

Religion generally serves as a spiritual and value system for human beings and guides people to understand the meaning of life and death. This study found that the adult children who reported practicing an Eastern religion, including Buddhism, Taoism, and Confucianism, have a higher opportunity of being classified as authoritarian FP, whereas those whose religion was Christianity have a higher opportunity to belong to reciprocal FP.

In the teaching of Buddhism, a good death is regarded as a great blessing to be pursued [42]. In practice, it suggests coherent concepts of FP, authoritarian FP in particular, by emphasizing that children should pay gratitude, reverence, and honor toward parents, as parents are their creators and educators [43,44]. Moreover, Confucianism has a philosophical stance among Chinese people and has shaped negative and pessimistic beliefs toward health events, especially when encountering an advanced disease, poor prognosis, or reaching end of life, by interpreting it as doom, punishment, or retribution for one’s wrongdoing [8]. If an advanced disease occurs in an older parent, Chinese adult children may insist to protect their parents from being informed about their terminal health condition, which is a typical representation of authoritarian FP.

In contrast, Western religions, such as Christianity, believe that God is the creator of human beings and the universe. Therefore, God, rather than parents, comes first among all beings and things [36]. Christians perceive illness as the plan of God and death is a reunion with God. They may perceive death as a fate associated with God’s will [45]. Both patients and their families probably feel it is easier to accept the disclosure of bad news about their health. In this case, it allows the open sharing of feelings and discussion of end-of-life planning among family members, and, accordingly, Christians in this study tended to be classified as practicing reciprocal FP.

Macao Chinese with a higher education level were more likely to practice reciprocal FP. Relatively more people in the sample were categorized as reciprocal FP, which may be attributed to the large proportion of the participants that received higher education. Those with higher education can be more competent in accessing appropriate means to satisfy the needs of the ill parents [36]. Moreover, education may enhance the probability of a better job, and those with a better job are likely to be rewarded with a better salary. Zhan et al. [25] claimed that actualization of filial duties, currently to some extent, requires considerable financial ability for paying medical bills, employing formal caregivers to take care of older parents, or affording a quality nursing home.

Furthermore, a higher preference for a good death was identified in this study as a significant attribute for reciprocal FP in adult Chinese of Macao. Chinese adult children with high reciprocal FP have higher motivation to emotionally and spiritually care for their parents and cultivate intimate parent–child relationships [2]. Such a relationship may allow deeper conversations between parents and children and sharing of views and wishes, including preferences for death arrangements. A good family relationship is also found to be a key element for a good death [46]. Regarding authoritarian FP, special attention in clinical treatment and care has to be paid to people with this trait, as they are used to oppress their own wishes and show lower self-esteem [2]. There is a potential for them to hesitate to express their wishes and discuss end-of-life planning with parents, rendering both the good death of parents and FP actualization of adult children unachievable.

### Limitations

Due to the employment of snowball and convenient sampling, the results could not be generalized to the whole Macao population. As self-administered questionnaires were used, participants were not able to ask questions regarding the research and the content of the questionnaire outside office hours (participants could contact the research team, as the contact information was provided on the informed consent page). This may have affected the valid response rate. In addition, web-based data collection might have resulted in a relatively high level of education among the participants in this study [47,48]. Future studies that employ different data collection channels are recommended. It is also suggested to further explore the association between the quality of end of life of parents and different FP representation of Chinese adult children.

## 5. Conclusions

This study is among the first to explore the filial representation at parents’ end of life of Chinese adults, as well as to identify the predictors of differentiating dual FP representations in such a context. Our findings indicated that more Macao Chinese were likely to practice reciprocal FP compared to authoritarian FP if they had to encounter parents’ end of life. Among all factors, older age and being religious were found to be strong predictors of conceptualizing authoritarian FP; higher education and higher preference for a good death were strong predictors of conceptualizing reciprocal FP. From a clinical perspective, clinicians could obtain a preliminary perception of the type of FP of Chinese patients and their families whom they care for based on the four predictors of FP representations. Such assessments could help clinicians determine an appropriate approach for the patients and their families to better understand the preference of a good death and gain respect from all members of the family. The provision of culturally sensitive treatment and care, as such, would address the best interest or final wish of a dying parent, resolve the conflict among patients, families, and clinicians, and minimize the moral distress of clinicians in all clinical settings.

## Figures and Tables

**Table 1:** Participant characteristics with correlation analyses to examine FPR differences (*n* = 705).

		Total (*n* = 705)		Authoritarian (*n* = 150)		Reciprocal (*n* = 555)			
Variables		*n*	%	*n*	%	*n*	%	*χ*^2^/*t*	*p*-Value
Gender								0.40	0.56
	Male	133	18.87	31	23.31	102	76.69		
	Female	572	81.13	119	20.80	453	79.20		
Age (year)								35.20	0.00 ^1^
	18–34	279	39.57	44	15.77	235	84.23		
	35–54	255	36.17	42	16.47	213	83.53		
	55–74	171	24.26	64	37.43	107	62.57		
Education level								47.34	0.00 ^1^
	High school or below	176	24.96	69	39.20	107	60.80		
	Bachelor	397	56.31	67	16.88	330	83.12		
	Master’s or above	132	18.72	14	10.61	118	89.39		
Marital experience								12.88	0.00 ^1^
	No	237	33.62	32	13.50	205	86.50		
	Yes	468	66.38	118	25.21	350	74.79		
Religious beliefs								23.06	0.00 ^1^
	None	400	56.74	69	17.25	331	82.75		
	Christianity	121	17.16	19	15.70	102	84.30		
	Buddhism/Taoism	184	26.10	62	33.70	122	66.30		
Participation of religious activities (*n* = 312)								0.35	0.84
	Never	76	24.36	18	23.68	58	76.32		
	Low	163	52.24	44	26.99	119	73.01		
	High	73	23.40	20	27.40	53	72.60		
Employment status								17.88	0.00 ^1^
	Not employed	203	28.79	64	31.53	139	68.47		
	Employed	502	71.21	86	17.13	416	82.87		
Children								15.30	0.00 ^1^
	None	296	41.99	42	14.19	254	85.81		
	Have children	409	58.01	108	26.41	301	73.59		
Sibling								2.05	0.22
	None	52	7.38	7	13.46	45	86.54		
	Have sibling(s)	653	92.62	143	21.90	510	78.10		
Living with parent(s)								0.55	0.50
	No	447	63.40	99	22.15	348	77.85		
	Yes	258	36.60	51	19.77	207	80.23		
Closeness with father (*n* = 692)								2.52	0.13
	Not close	322	46.53	59	18.32	263	81.68		
	Close	370	53.47	86	23.24	284	76.76		
Closeness with mother (*n* = 694)								0.10	0.83
	Not close	177	25.50	36	20.34	141	79.66		
	Close	517	74.50	111	21.47	406	78.53		
Father alive								7.26	0.01 ^1^
	No	258	36.60	69	26.74	189	73.26		
	Yes	447	63.40	81	18.12	366	81.88		
Mother alive								28.95	0.00 ^1^
	No	158	22.41	58	36.71	100	63.29		
	Yes	547	77.59	92	16.82	455	83.18		
Father’s death experience (*n* = 258)								0.63	0.42
	Good	62	24.03	19	30.65	43	69.35		
	Not good/neutral	196	75.97	50	25.51	146	74.49		
Mother’s death experience (*n* = 158)								0.01	1.00
	Good	58	36.71	21	36.21	37	63.79		
	Not good/neutral	100	63.29	37	37.00	63	63.00		
Self-rated health								4.07	0.05 ^1^
	Good	357	50.64	65	18.21	292	81.79		
	Not good	348	49.36	85	24.4	263	75.57		
GDI, *M* (*SD*)		305.48	26.17	303.37	26.40	306.05	26.10	−1.11	0.27

^1^ *p*-value < 0.05.

**Table 2:** Results of FPR-EoL Scale Items (*n* = 705).

Item	Min.	Max.	*M*	*SD*
Authoritarian FP representations				
1. I would choose to temporarily leave my job and concentrate on accompanying and caring for my parents	1	5	3.67	1.16
3. I would ask medical staff not to disclose the illness to my parents in order to protect them	1	5	2.77	1.29
5. When discussing illness with my parents, I would only report good news rather than bad news	1	5	3.07	1.26
8. I would definitely seek medical advice from a number of doctors for my parents, believing that miracles may happen	1	5	3.56	1.18
10. I would commit to living up to my parents’ expectations, even if I am unwilling or unable to do so (e.g., getting married, having children, moving back home etc.)	1	5	3.42	1.19
12. Even if I do not agree to parents’ instructions about funeral arrangements, I would carry out them according to their wishes	1	5	4.29	0.74
13. I would protect dignity of my parents and demand others’ respect for them	1	5	4.47	0.75
14. I demand obedience from other family members (spouses, children, siblings, etc.) to my parents	1	5	3.90	0.98
16. I would sign the consent of Do Not Resuscitate (no cardiopulmonary Resuscitation) according to doctor’s opinion	1	5	3.85	1.03
18. I would follow customs and arrange a grand funeral for parents	1	5	3.29	1.15
Subtotal of authoritarian	21	50	36.29	5.36
Reciprocal FP representations				
2. If I could not accompany my parents every day, I would inform them to seek their understanding	1	5	4.50	0.77
4. If doctors say that the treatments are no longer able to improve parents’ condition, I would consider talking with my parents about stopping the treatments	1	5	3.49	1.20
6. I would take the initiative to talk about death with parents	1	5	3.54	1.22
7. I would help my parents to prepare for the coming of death (e.g., encourages expression of emotions, accompany, listen to them etc.)	1	5	4.17	0.95
9. I would ease physical pain and suffering of my parents, instead of allowing tolerance of pain caused by the illness and treatment, and life extension	1	5	4.02	1.08
11. I would try my best to appease my parents’ worries about future living of other family members	1	5	4.47	0.61
15. I would actively ask parents about their inner feelings, thoughts, and needs	1	5	4.33	0.79
17. I would let parents spend the rest of their lives at where they like	2	5	4.40	0.62
19. I would let parents die at a place of their choice	1	5	4.30	0.67
Subtotal of reciprocal	19	45	37.22	4.18
Total	43	94	73.50	7.57

**Table 3:** Perception of good death of participants (n = 705).

Domains	Min.	Max.	*M*	*SD*	Ranking
Total	183	361	305.48	26.17	
1. Physical and psychological comfort	4	21	17.99	3.09	10
2. Dying in a favorite place	7	21	18.10	2.85	9
3. Maintaining hope and pleasure	7	21	18.27	2.50	6
4. Good relationship with medical staff	9	21	18.78	2.28	4
5. Not being burden to others	5	21	18.67	2.73	5
6. Good relationship with family	6	21	19.07	2.16	2
7. Independence	4	21	17.63	3.68	12
8. Environmental comfort	6	21	18.25	2.51	7
9. Being respected as an individual	9	21	19.14	2.04	1
10. Life completion	3	21	18.11	2.86	8
11. Receiving enough treatment	3	21	15.80	3.53	15
12. Natural death	7	21	17.69	2.80	11
13. Preparation for death	6	21	19.04	1.96	3
14. Control over the future	6	21	17.08	3.00	13
15. Unawareness of death	3	21	10.21	4.23	17
16. Pride and beauty	3	21	9.61	3.81	18
17. Feeling that one’s life is worth living	7	21	16.85	2.68	14
18. Religious and spiritual comfort	3	21	15.18	3.91	16

## Data Availability

Data available on request due to ethical considerations. The data presented in this study are available on request from the corresponding author.
